# Trends in Outcomes and Hospitalization Charges among Mechanically Ventilated Patients with Myasthenia Gravis in the United States

**Published:** 2009-09

**Authors:** Nizar Souayah, Abu Nasar, M. Fareed K. Suri, Jawad F. Kirmani, Mustapha A. Ezzeddine, Adnan I. Qureshi

**Affiliations:** *Department of Medicine, University of Medicine and Dentistry of New Jersey, Newark, New Jersey, USA; Zeenat Qureshi Stroke Research Center, University of Minnesota, Minneapolis, MN, USA*

**Keywords:** myasthenia gravis, myasthenia crisis, mechanical ventilation, cost, outcome, length of stay, mortality

## Abstract

**Introduction:**

To assess the impact of new therapeutic strategies on outcome and cost of hospitalization among patients with myasthenia gravis (MG) who are mechanically ventilated in United States.

**Methods:**

Using a retrospective analysis of cross sectional survey, we determined the rates of occurrence, in-hospital outcomes, and mean hospital charges for patients hospitalized with MG requiring mechanical ventilation in 1991–1992 using the Nationwide Inpatient Survey (NIS) and compared these outcomes with homologous data from 2001–2002. NIS is the largest all-payer inpatient care database in the United States.

**Results:**

When comparing data from 2001–2002 with data from 1991–1992, we found a higher number of admissions for MG that required mechanical ventilation (994 vs. 652). The proportion of women was similar (53% vs. 60%). The average age (in years ± standard deviation) was significantly higher (65 ± 17 vs. 58 ± 18, *p*=0.0002). The length of hospitalization (in days ± standard deviation) was not different (22 ± 19 vs. 21 ± 16). Discharge to home occurred less frequently (29% vs. 60%, *p*=0.0001) and in hospital mortality minimally lower (13% vs. 15%). There was a significant increase in mean hospital charges ($118,000 vs. $84,100 adjusted for inflation, *p*=0.0001). In hospital mortality was higher among urban teaching hospitals compared with urban non teaching hospitals in 2001–2002.

**Conclusions:**

Despite improvement in therapeutic strategies from 1991 to 2002, there was only a modest reduction in mortality and no substantial reduction of length of hospitalization for patients with MG requiring mechanical ventilation.

## INTRODUCTION

Myasthenia gravis (MG) is an acquired, immune mediated disease that affects the neuromuscular junction at the postsynaptic level (1, 2). Myasthenia crisis, defined as an acute weakness of respiratory and bulbar muscles requiring ventilatory assistance, occurs in 15–20% of MG patients (3). The condition can be life threatening because of respiratory failure and other associated complications (3–5). The advance in understanding the pathogenesis of MG has led to the development of new effective treatments and improved methods of pulmonary support and mechanical ventilation (6–13). This progress led to the reduction of MG associated mortality from 40% in 1960 to approximately 4% in 1990 (3). However, national estimates of the impact of these new therapeutic strategies on hospital outcomes and cost of mechanically ventilated patients with MG crisis are not estimated. Our current understanding of complications, hospitalization costs and outcomes of MG crisis is based on a body of literature from single-center studies of 1970 and early 1980 (4, 5, 14, 15) which was extended by a few retrospective studies from 1990 and the early 2000's after the advent of specific intensive care units, the widespread use of immunotherapies, and improved nursing care (3, 6, 16, 17). Due to limitations in existing reports, we performed this study using nationally representative data to assess the impact of new therapeutic strategies on outcome and cost of hospitalization among patients with MG who are mechanically ventilated.

## DESIGN/METHODS

We used the data from the Nationwide Inpatient Survey (NIS) sponsored by the Agency for Healthcare Research and Quality (AHRQ) (18–21). The NIS is the largest inpatient care database in the United States designed to identify, track, and analyze national trends in health care utilization, access, charges, quality, and outcomes. The NIS is the only national hospital database with charge information on all patients, regardless of payer, including patients covered by Medicare, Medicaid, and private insurance, and those who are uninsured. Detailed information on the design of the NIS is available at http://www.hcup-us.ahrq.gov. We identified adult (age >20 years) patients using the International Classification of Disease, 9^th^ Revision, Clinical Modification (ICD-9-CM) code for primary diagnosis of MG (358.0) and either primary or secondary procedure codes for continuous mechanical ventilation [967, 967.0, 967.1, 967.2].

During the first period, we looked at 1991–1992, when none of the new treatment modalities for MG exacerbations were available, whereas by 2001–2002 several strategies were widely available including specialized neuro-intensive care units and effective immunotherapies. We determined the rates, hospital outcomes, and charges incurred among patients hospitalized with MG requiring mechanical ventilation in 1991–1992 and compared these variables among those hospitalized in 2001–2002. The variables analyzed were: age, sex, race/ethnicity, length of stay, discharge status (categorized into routine, home health care, short-term hospital, other facility including intermediate care and skilled nursing home, or death), and total hospitalization charges. We used the SUDAAN (22) software program to convert raw counts generated from the NIS database into weighted counts that we used to generate national estimates. We used the chi-square test for categorical data and analysis of variance for continuous data to detect any significant differences between 1991–1992 and 2001–2002 and applied the Bonferroni adjustment when multiple comparisons were made. Because differences in hospital charges between the two periods may be related to inflation, we adjusted for increase in cost expected from inflation. We used the data from the Consumer Price Index (CPI) too adjust for average change in prices related to inflation (http://www.bls.gov/cpi/). We also evaluated the association between various patient outcomes and the hospitals in which they were treated (rural, urban nonteaching, and urban teaching hospitals). The definitions are as follows: urban hospital-located in a metropolitan statistical area; and teaching hospital with American Medical Association approved residency program and either membership in the Council of Teaching Hospitals or a ratio of full-time equivalent interns and residents to beds of 0.25 or higher.

## RESULTS

### Demographic and clinical characteristics

The demographic and clinical characteristics of mechanically ventilated MG patients are shown in Table 1 and Table 2. There was a 53% increase in the number of admissions for MG requiring mechanical ventilation in 2001–2002 compared with 1991–1992. Women were more frequently admitted with MG requiring mechanical ventilation in both time periods. The racial/ethnic distribution of patients was similar in both time periods with predominance of white patients. The mean age (years ± standard deviation) was higher among patients hospitalized in 2001–2002 compared with those hospitalized in 1991–1992 (65 ± 17 vs. 58 ± 18, *p*=0.0002).

### In-hospital outcomes

The length of hospitalization (days ± standard deviation) in both time periods was similar: 22 ± 19 in 2001–2002 and 21 ± 16 in 1991–1992. In hospital mortality was 13% in 2001–2002 and 15% in 1991–1992. Discharge to home was lower (29%) in 2001–2002, compared with 1991–1992 (60%), however, transfer to skilled nursing and an intermediate facility was increased (37% vs. 9%).

### Total hospitalization charges

The mean charges for hospitalization of each patient, after adjustment for inflation, increased by 51% in 2001–2002 compared with 1991–1992 ($118,000 versus $84,100, *p*=0.0001). The average daily hospitalization charges per patient (inflation-adjusted) were $5500 and $3932, respectively.

### Subgroups Analysis by age

We compared the demographic and outcome characteristics of mechanically ventilated MG patients under the age of 50 years with those 50 and older (Table 1). There was a substantial increase in the number of patients over 50 years of age in 2001–2002 (80% vs. 62%, *p*=0.0001). In patients aged >50 years, 20% were discharged home in 2001–2002 vs. 54% in 1991–1992. Their average length of stay was 22 days ± 19 in 2001–2002 vs. 22 ± 15 in 1991–1992 (*p*=0.7855). In neither age group was there a significant decrease in the rate of in hospital mortality in 2001–2002 compared to 1991–1992.

**Table 1:** Demographic characteristics and in-hospital outcomes among myasthenia gravis patients requiring mechanical ventilation in the United States (Nationwide Inpatient Survey, 1991–1992 and 2001–2002)

Characteristics	MG patients 1991–1992			MG patients 2001–2002		
	Total	Patients aged less than 50 years	Patients aged 50 years or greater	Total	Patients aged less than 50 years	Patients aged 50 years or greater

Total	651	247	404	994	196	798
Age (year ± SD)	58 ± 18^a^	38 ± 8	69 ± 10	65 ± 17	39 ± 9	72 ± 10
Gender						
Men	259 (40%)	88 (36%)	171 (42%)	464 (47%)	71 (36%)	393 (49%)
Women	392 (60%)	159 (64%)	233 (58%)	530 (53%)	125 (64%)	405 (51%)
Discharge Status						
Home	387 (60%)^a^	173 (70%)	214 (54%)	284 (28%)	126 (64%)	158 (20%)
Transfer to short term Hospital	57 (9%)	23 (9%)	34 (8%)	108 (11%)	15 (8%)	93 (12%)
Other transfer, Including skilled nursing facility, intermediate care	61 (9%)^a^	14 (6%)	47 (12%)	377 (37%)	16 (8%)	361 (45%)
Home health care	41 (6%)	15 (6%)	26 (6%)	94 (9%)	34 (17%)	60 (8%)
Died in Hospital (Mortality)	100 (15%)	22 (9%)	78 (20%)	125 (13%)	5 (3%)	120 (15%)
Not stated/Missing	5 (1%)	0 (0%)	5 (1%)	6 (1%)	0 (0%)	6 (1%)
Length of stay (days)	21 ± 16	20 ± 17	22 ± 15	22 ± 19	18 ± 20	22 ± 19
Total hospitalization charges ($)	66,300^a^ (Inflation adjusted cost 84,100)	67,700 (p=0.091)	65,400	118,000	113,100	119,100

a *p*<0.05 by comparison between patients admitted in 1991–1992 and 2001–2002. SD, standard deviation.

### Subgroup analysis by type of hospital

In urban teaching hospitals, there was significant increase in the mean age (years ± standard deviation) of patients admitted in 2001–2002 compared with those admitted in 1991–1992 (65 ± 17 vs. 55 ± 18, *p*=0.0001). However, this trend was not seen in urban nonteaching hospitals (65 ± 16 vs. 60 ± 18, p = 0.13). In both time periods, the urban teaching hospitals had a longer average length of stay, and higher mortality (Table 2). For urban teaching hospitals, cost of treatment did not change much over the ten-year period ($75,000 in 2001–2002 and $74,500 in 1991–1992), but the nonteaching hospitals had a prominent increase ($118,300 in 2001–2002, vs. $58,000 in 1991–1992). In rural hospitals, 72 patients were hospitalized in 2001–2002 and only 10 patients were hospitalized in 1991–1992 limiting any conclusive analysis.

**Table 2:** The demographic and outcome characteristics of myasthenic crisis patients admitted to urban teaching hospitals and urban nonteaching hospitals in the United States (Nationwide Inpatient Survey, 1991–1992 and 2001–2002)

Characteristics	1991–1992		2001–2002	
	Urban teaching 346	Urban non-teaching 296	Urban teaching 596	Urban non-teaching 325

Age (years)	55 ± 18	60 ± 18	65 ± 17	65 ± 16
Sex				
Male	32%	47%	53%	36%
Female	68%	53%	47%	64%
Race				
White	30%	60%	54%	54%
African-American	6%	6%	10%	12%
Not stated/missing	60%	26%	26%	21%
Length of stay (days)	23 ± 15	20 ± 16	24 ± 20	18 ± 17
Discharge status				
Routine	70%	48%	27%	31%
Transfer to short term hospital	2%	15%	5%	18%
Other transfer, including skilled nursing facility, intermediate care	6%	14%	38%	41%
Home health care	3%	10%	13%	3%
Against medical advice	0%	0%	0%	0%
Died in hospital (mortality)	18%	12 %	16%	7%
Not stated/missing	0%	1.7%	0.6%	0%
Total hospital charges ($)	74,500	58,000	75,200	118,300

## DISCUSSION

### Salient findings of the study

Over a span of ten years (1991–1992 to 2001–2002), the following trends were revealed:

- Older patients are being admitted with MG and requiring mechanical ventilation;
- In hospital mortality and length of hospitalization demonstrated minimal changes;
- Despite adjusting for inflation, mean hospital charges demonstrated a significant increase;
- The reduction in hospital mortality became more prominent in patients under the age of 50 years;
- More patients with MG requiring mechanical ventilation were admitted to urban teaching hospitals throughout the time period, but their differential admission rates became more prominent in 2001–2002;
- The mortality reduction was more prominent in urban non teaching hospitals than in urban teaching hospitals;
- There is a suggestion that, despite an increase in hospitalization charges, minimal benefit was seen in reducing in hospital mortality and length of stay. This lack of benefit may be attributable to a higher proportion of older patients admitted in 2001–2002 with a higher likelihood of poor outcomes;
- There appeared to be an increase in proportion of MG inpatients admitted to urban teaching hospitals. Given the reduction in in-hospital mortality in urban non teaching hospitals, it may be presumed that patients with more severe disease were being selectively referred to urban teaching hospitals.

### Comparison with previous studies

With improved treatment strategies, the mortality rate had dramatically reduced in the past 40 years. It fell from 43% in mid 1950s to 4.5% in 1979 (4, 23–25). In a retrospective study over the decade from 1969 to 1978, Ferguson *et al.* (24) examined the outcome in thirty-one patients with myasthenia gravis who developed ventilatory failure and reported a mortality rate of 26%, improving from 70% mortality in a similar group of patients reported from the same facility for the years 1960–1968. In another retrospective study performed at the Mayo Clinic in 1978–1979, only one death was reported among 22 patients with myasthenia crisis (5). In another retrospective study performed at Columbia Presbyterian hospital, between 1983 and 1994, 6% of patients with MG crisis died during crisis and another four patients after extubation (total death rate 13%) (3). The mortality in that study was similar to the mortality observed in our 2001–2002 admissions. In a more recent retrospective study performed at the Johns Hopkins hospital that included 18 patients with myasthenia crisis from 1990 to 1998, one death was reported (6%) (17). The mortality in our analysis was higher than that observed in reports from these specialized centers. It is possible that there may be a continued reduction of in-hospital mortality in selected hospitals which is not represented at a national level.

In our subgroup of patients below 50 years age, the death rate was lower than in the Johns Hopkins and Columbia Presbyterian studies (3, 17). Over the age of 50 years, our death rate was higher that the Johns Hopkins and Columbia Presbyterian studies where the average patient age was 52 and 55 years, respectively. In our 2001–2002 admissions, the mortality in urban teaching hospitals (16%) was higher than in urban nonteaching hospitals (7%). In our 1991–1992 group, the mortality in urban teaching hospitals was 18.5% whereas it was 12% in urban nonteaching hospitals. Both mortalities were higher than the corresponding mortalities in 2001–2002 despite the increase in average age of patients admitted to the urban teaching hospitals.

The average length of hospitalization in our study was 21 days in 1991–1992 and 22 days in 2001–2002. The average hospitalization was 35 days in the Columbia Presbyterian study (3) and 17 days in the Johns Hopkins study (17). In the absence of knowledge regarding baseline severity of disease, it is difficult to compare these values appropriately. In urban teaching hospitals and urban nonteaching hospitals, there was no significant difference in hospital length of stay between the 1991–1992 and 2001–2002 groups. The total hospital charges were higher in urban non teaching hospitals admissions compared with urban teaching hospital admissions, despite a shorter length of stay in urban non teaching hospitals It is possible that the presence of more specialized neuro ICU units in academic hospitals helped managing patients in a more cost effective manner.

### Potential explanations for the observed findings

Initially considered as a disease of young adults (26), MG was later found to be a disease of younger women with female-to-male ratio of 2:1 and older men in whom peak age of onset is after the age of 60 years (27–29). This concept was challenged by several recent studies reporting a substantial number of patients in whom the disease occurred much later in life (29–33). In the past decade, the incidence of MG has increased from up to 5 per million (34) to up to 21 cases per million (30, 31, 35) mainly from increased incidence of late onset MG. The prevalence of MG increased over time compared to other immunological disorders (31, 36) especially in patients older than 50 years. This increase is probably related to the availability of more effective treatment of MG leading to accumulation of patients with early onset MG who live longer and a substantial increase of patients who develop the disease later in life (37). It is estimated that 61% of MG patients in United States are older than 50 years (38). This is reflected in our study by the 52% increase in the number of patients over 50 and increase in the mean age by 7.5 years over the last decade.

Late onset myasthenia, defined by onset after the age of 50 years, has specific epidemiological, immunological and clinical characteristics (29, 35). It is gender independent and has more severe clinical pattern than the early onset form of MG (29, 35, 39). Its treatment can be challenging because of frequent resistance to acetyl cholinesterase inhibitors, poor response to thymectomy, vulnerability to plasma exchange, and long term steroids administration (29, 40). Immunologically, patients with late onset MG have less incidence of thymoma, lower concentration of acetylcholine receptors antibodies and high incidence of antibodies to striated muscles antigens (29, 33, 35). Routine discharge occurred in only 28% of patients in our 2001–2002 group whereas it occurred in 60% in 1991–1992 suggesting that MG requiring mechanical ventilation become a more disabling disease. However, this could also be explained by the presence of lower severity of disease in the 1991–1992 admissions. The NIS data base does not provide the data regarding MG severity score. It is possible that the increased discharges with status of transferring to short term hospital, skilled nursing facility, and intermediate care may be due to increased availability of the such care facilities in 2001–2002 than in 1991–1992. The progress in ICU management of MG in the past decade may have resulted in higher utilization of non-invasive assisted ventilation methods in 2001–2002. Therefore, mechanical ventilation may be reserved for patients with more severe MG manifestations resulting in an apparently higher rate of disability and cost, and obscuring the mortality benefit in 2001–2002. In addition, the increase in the number of late onset myasthenia gravis and the number of elderly myasthenic patients due to greater survival may have increased the proportion of elderly patients with more severe myasthenia and more comorbid conditions. This also may explain the increase in total hospitalization charges betweenn 2001–2002 and 1991–1992 despite the fact that the length of hospitalization in both time periods was similar.

It is well known that in less than 5% of MG patients, no progress in their treatment have been achieved since the introduction of immunosuppressive medication with frequent MG crisis and increased morbidity and mortality (7, 41). With the increase in the incidence of late onset MG in the past decade and the aging of early onset MG patients, the prevalence of MG resistant to therapeutic intervention may be higher than 5% of total MG patients. This may in part explain the modest reduction in mortality despite the increase in the length and cost of hospitalization. However, differences in comorbid conditions and baseline severity of MG may explain some of the differences in cost and outcome observed in our study.

## CONCLUSION

In summary, a modest improvement in mortality and no substantial change in length of stay have been observed among MG patients requiring mechanical ventilation in 2001–2002 compared with 1991–1992 despite a dramatic increase in hospitalization charges. This may be explained by higher proportion of older patients with MG admitted in recent years. The increase in prevalence of late onset MG and the aging of early onset MG patients has also increase the prevalence of MG patients less responsive to standard treatments. We believe that better understanding of national trends will allow for appropriate resource allocation among patients with MG.
